# Seaweed fails to prevent ocean acidification impact on foraminifera along a shallow-water CO_2_ gradient

**DOI:** 10.1002/ece3.1475

**Published:** 2015-03-31

**Authors:** Laura R Pettit, Christopher W Smart, Malcolm B Hart, Marco Milazzo, Jason M Hall-Spencer

**Affiliations:** 1 Marine Biology and Ecology Research Centre, School of Marine Science and Engineering, Plymouth University, Drake CircusPlymouth, PL4 8AA, U.K; 2 School of Geography, Earth and Environmental Sciences, Plymouth University, Drake CircusPlymouth, PL4 8AA, U.K; 3 DiSTeM University of Palermo, CoNISMavia Archirafi 28, 90123, Palermo, Italy

**Keywords:** Benthic foraminifera, blue carbon, coastal communities, ocean acidification, shallow-water CO_2_ seeps

## Abstract

Ocean acidification causes biodiversity loss, alters ecosystems, and may impact food security, as shells of small organisms dissolve easily in corrosive waters. There is a suggestion that photosynthetic organisms could mitigate ocean acidification on a local scale, through seagrass protection or seaweed cultivation, as net ecosystem organic production raises the saturation state of calcium carbonate making seawater less corrosive. Here, we used a natural gradient in calcium carbonate saturation, caused by shallow-water CO_2_ seeps in the Mediterranean Sea, to assess whether seaweed that is resistant to acidification (*Padina pavonica*) could prevent adverse effects of acidification on epiphytic foraminifera. We found a reduction in the number of species of foraminifera as calcium carbonate saturation state fell and that the assemblage shifted from one dominated by calcareous species at reference sites (pH ∼8.19) to one dominated by agglutinated foraminifera at elevated levels of CO_2_ (pH ∼7.71). It is expected that ocean acidification will result in changes in foraminiferal assemblage composition and agglutinated forms may become more prevalent. Although *Padina* did not prevent adverse effects of ocean acidification, high biomass stands of seagrass or seaweed farms might be more successful in protecting epiphytic foraminifera.

## Introduction

Ocean acidification is, primarily, caused by anthropogenic emissions of CO_2_. A third of these recent emissions have dissolved into water at the ocean surface causing mean pH to fall by 0.1 pH units since pre-industrial times and this is predicted to decrease by a further 0.3–0.4 units by the end of this century (IPCC 2014). These changes in seawater carbonate chemistry are detrimental to most of the organisms that have been studied so far, but benefit others, causing profound changes in coastal ecosystems (Hall-Spencer et al. 2008; Kroeker et al. 2013). The effects of ocean acidification are a major concern as rapid shoaling of the calcium carbonate saturation horizon is exposing vast areas of marine sediment to corrosive waters worldwide (Feely and Chen 1982; Olafsson et al. 2009).

The geological record shows that the present rate of ocean acidification is likely to be faster than at any time in the last 300 million years (Zachos et al. 2005; Hönisch et al. 2012). Foraminifera (single-celled protists) occur from coasts to the deep sea (Goldstein 1999) and some even live in freshwater, or terrestrial environments. Their fossil record dates back to the beginning of the paleozoic (Murray 1979) and provides precious insights into past fluctuations in seawater carbonate chemistry. Deep-sea foraminifera suffered extinctions during periods of high CO_2_ in the past, such as during the Paleocene–Eocene Thermal Maximum (PETM) (Hönisch et al. 2012).

Laboratory investigations have shown that the shell weight of planktonic foraminifera declines as seawater calcium carbonate saturation falls (Bijma et al. 1999). Spero et al. (1997) found that *Orbulina universa* d’Orbigny shell weight increased by 37% when grown at high *vs*. background calcium carbonate levels. Bernhard et al. (2009a,b) showed that deep-sea calcareous foraminifera are killed by direct exposure to injected CO_2_, whereas thecate and agglutinated species survive. Acidification of seawater with 1000 ppm CO_2_ caused shell dissolution in the shallow-water calcified foraminifera *Haynesina germanica* (Ehrenberg), and their ornamentation used for feeding was reduced and deformed (Khanna et al. 2013). Some laboratory experiments, however, have found calcareous foraminifera not to be negatively impacted by ocean acidification (Hikami et al. 2011; Vogel and Uthicke 2012), or the response to be complex, with an initial positive response, up to intermediate *p*CO_2_, followed by a negative response (Fujita et al. 2011; Keul et al. 2013). In a review of 26 studies that have examined foraminiferal response patterns to carbonate chemistry, Keul et al. (2013) found that three reported a positive response to increased *p*CO_2_ and three reported no effect.

Carbon dioxide seeps create gradients of calcium carbonate saturation that provide opportunities to examine the long-term effects of corrosive waters on calcified marine life (Boatta et al. 2013; Milazzo et al. 2014). There are steep reductions in species richness around CO_2_ seeps; calcified foraminifera are intolerant of chronic exposure to acidified waters in coastal sediments of the Mediterranean Sea (Dias et al. 2010) and off Papua New Guinea (Fabricius et al. 2011; Uthicke et al. 2013).

Geo-engineering options are being considered as the far-reaching effects of ocean acidification are predicted to impact food webs, biodiversity, aquaculture, and hence society (Williamson and Turley 2012). Seaweeds and seagrasses can thrive in waters with naturally high *p*CO_2_ (Martin et al. 2008; Porzio et al. 2011), so could potentially provide an ecologically important and cost-effective means for improving seawater conditions for sensitive organisms by consuming dissolved CO_2_ and raising local seawater pH (Gao and Mckinley 1994; Manzello et al. 2012; Chung et al. 2013; Hendriks et al. 2014). The primary production of those seagrasses and macroalgae that are carbon limited increases as CO_2_ levels rise (Connell and Russell 2010; Manzello et al. 2012). A blue carbon project has been established in South Korea, where natural and made-made marine communities are being used to remove CO_2_ in coastal regions. The higher the biomass of these plant communities, the more CO_2_ is drawn down (Chung et al. 2013).

Here, we assess whether a common Mediterranean seaweed (*Padina pavonica* (Linné) Thivy) can create local ocean acidification sanctuaries by providing refugia for calcification. This species was chosen as many heterokont algae, and *Padina* spp. in particular, are resilient to the effects of ocean acidification (Porzio et al. 2011; Johnson et al. 2012). In addition, *Padina* is abundant and hosts calcified foraminiferal epiphytes at our study sites (Langer 1993). Although benthic foraminifera living within and on sediment are expected to be adversely affected by ocean acidification, epiphytic foraminifera may be protected from the detrimental effects of ocean acidification, as macroalgal photosynthesis raises seawater pH (Cornwall et al. 2013). Photosynthesis by macroalgae utilizes the dissolved inorganic carbon (DIC) pool, usually in the form of 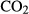 (Gao and Mckinley 1994). The surfaces of organisms have a microlayer (known as a diffusion boundary layer, DBL) that usually differs from the surrounding seawater chemistry (Hurd et al. 2011). Within the DBL, the chemistry is altered by metabolic processes, and in macroalgae, photosynthesis increases the pH in the daytime (Hurd et al. 2011; Cornwall et al. 2013). These DBLs are on the scale of micrometers to millimeters, but within macroalgal canopies, where there is reduced flow, larger concentration gradients (up to 68 mm) can develop (Cornwall et al. 2013).

Although macroalgae raise pH during the day, at night, pH in the DBL may drop to ∼7.8 in slow water flow (Cornwall et al. 2013). The macroalgae will still respire, but there will be no photosynthesis in the dark. Thus, epiphytes can experience a wide pH range on a daily basis, which exceeds open water mean pH changes expected due to ocean acidification (Cornwall et al. 2013). Some foraminifera host algal symbionts which can affect the pH of the DBL around the foraminiferal test (Köhler-Rink and Kühl 2000). Köhler-Rink and Kühl (2000) found that under saturating light conditions photosynthetic activity of the endosymbiotic algae increased pH up to 8.6 at the test surface. In the dark, pH at the test surface was lowered relative to the ambient seawater of pH 8.2.

In laboratory experiments, it has been found that algal epiphytes can resist levels of acidification associated with *p*CO_2_ values of 1193 ± 166 *μ*atm (Saderne and Wahl 2013). In situ experiments show that seagrass photosynthesis can enhance calcification of the calcareous red algae *Hydrolithon* sp. 5.8-fold (Semesi et al. 2009). There are limits, however, to the role that marine plants can play in buffering the effects of ocean acidification. Martin et al. (2008) found a dramatic reduction in calcareous epiphytes on seagrass blades as pH reduced below a mean pH 7.7 near to CO_2_ seeps off Ischia in the Mediterranean.

**Figure 1:**
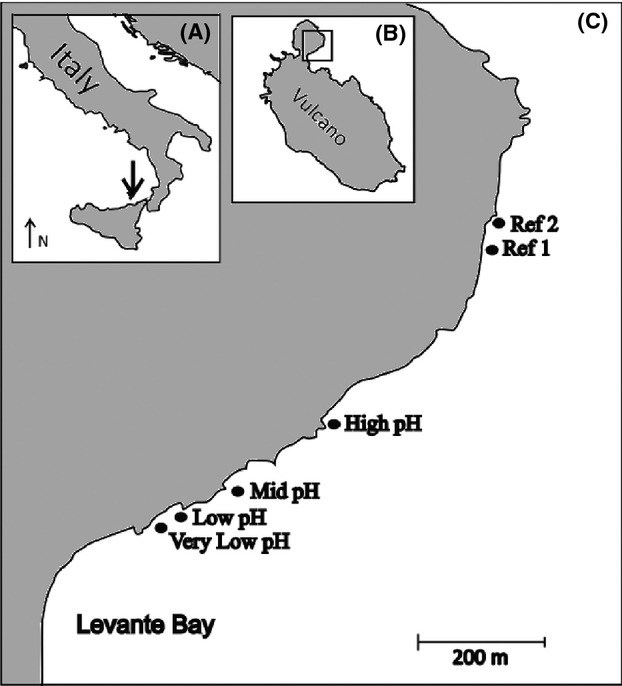
Study area. (A) Italy with arrow marking Vulcano Island, part of the Aeolian islands chain, northeast Sicily, (B) Vulcano Island, (C) Location of sample sites. Very low pH is 38°25′9″ N, 14°57′38″E, Ref 2 is 38°25′20″N, 14°58′3″E.

We test the hypothesis that the brown seaweed *P. pavonica* collected along a CO_2_ gradient off Vulcano Island, Italy (Fig.1), provides a refuge for benthic foraminifera along a gradient of overlying seawater acidification. Foraminifera play an important role in the Earth’s CO_2_/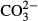 budget (Lee and Anderson 1991; Langer et al. 1997), so their response to ocean acidification may have important consequences for inorganic carbon cycling. A major foraminiferal die-off may act as a negative feedback on atmospheric CO_2_ levels and lead to a reduction in globally precipitated calcium carbonate (Dissard et al. 2010). The foraminifera may reflect responses of other small calcified animals, such as larval bivalves; therefore, if macroalgae are found to work well in limiting the effects of ocean acidification, people who rely on shellfish fisheries and aquaculture could grow seaweed to mitigate the effects of ocean acidification.

## Materials and Methods

Sampling took place near shallow submarine CO_2_ seeps in Levante Bay off Vulcano in the Mediterranean Sea, an island ca. 25 km northeast of Sicily (Fig.1). Our sites were chosen along part of a pH gradient where seawater was at ambient temperature and alkalinity, but was not affected by H_2_S (Boatta et al. 2013). Although variable, but small (∼400 ppm), amounts of H_2_S were found directly over the degassing area (Boatta et al. 2013), it should be noted that samples were not taken directly over the degassing area. Boatta et al. (2013) report that the sampling gradient used in the present study lacks toxic compounds such as H_2_S. Dissolved sulfide was below the detection limit (i.e., <15 *μ*mol/kg) at 5 m distance from the degassing area (Boatta et al. 2013). *Padina pavonica* thalli were collected from six sites in May 2012 (Fig.1). Thalli were collected from approximately 1 m water depth, by cutting algal blades above the sediment surface and placing into labeled plastic sample bags underwater following the methods of Langer (1993). Five replicates were collected from each site. The thalli were placed in aluminum trays and left to air dry, then 2 g of dry thallus from each sample was randomly selected and examined under a stereo-binocular microscope. Epiphytic foraminifera were removed and placed on micropalaeontological picking slides. Foraminifera were identified to species level where this was possible using, for example, Cimerman and Langer (1991) and Milker and Schmiedl (2012), and then counted. The aim was to examine at least 300 individuals from each sample as 300 individuals are believed to be statistically representative of the whole sample (Pielou 1966). This, however, was not possible in all cases due to the low number of foraminifera found in the low-pH sites. It should be noted that as the thalli were dried, allogromiid foraminifera were not examined during this study.

Although the carbonate chemistry conditions along the *p*CO_2_ gradient at Vulcano have been reported extensively by others (Johnson et al. 2012, 2013; Boatta et al. 2013; Vizzini et al. 2013; Kerfahi et al. 2014; Milazzo et al. 2014), additional samples were collected during three separate fieldwork campaigns between May 2011 and May 2013. At each of the sample sites, pH, temperature, and salinity were recorded using a calibrated YSI (556 MPS) pH (NBS scale) meter. The NBS scale has a precision error of around ±0.05 units (Dickson et al. 2007), which was considered acceptable given the >1 unit fluctuations across the study gradient.

Three replicate total alkalinity (TA) samples were collected from each site and 0.02% by volume of mercuric chloride was added to each. The samples were sealed and stored in the dark until analysis using an AS-ALK2 Total Alkalinity Titrator (Apollo SciTech Inc., Bogart, GA, USA), calibrated using total alkalinity standards (Dickson Laboratory, batch 121, Scripps Institution of Oceanography, California, USA). The pH, TA, salinity, and temperature were used to calculate the remaining carbonate chemistry parameters using CO2SYS (Lewis and Wallace 1998).

Some foraminifera were examined under a JEOL JSM 5600 LV SEM with a digital imaging system to aid taxonomic identification and determine whether there was any dissolution. Individuals were mounted on aluminum SEM stubs and sputter coated in an Emitech K550 gold sputter coater. Dissolution was noted if foraminifera tests showed etching, pitting, fragmentation, or enlarged pores. Test walls were examined at a higher magnification (×4000) in 11 of the individuals.

Raw foraminifera community assemblage data were used to calculate Shannon–Wiener diversity, Fisher alpha, and Pielou’s evenness indices which were analyzed using one-way ANOVA and Kruskal–Wallis tests. Nonmetric multidimensional scaling (nMDS) was used to examine assemblage shifts based on square root-transformed data to down-weight the contribution of dominant taxa. An ANOSIM test was conducted to test for similarities between sites, and a SIMPER test was used to determine discriminating species using PRIMER v6 (PRIMER-E Ltd., Ivybridge, UK).

## Results

**Figure 2:**
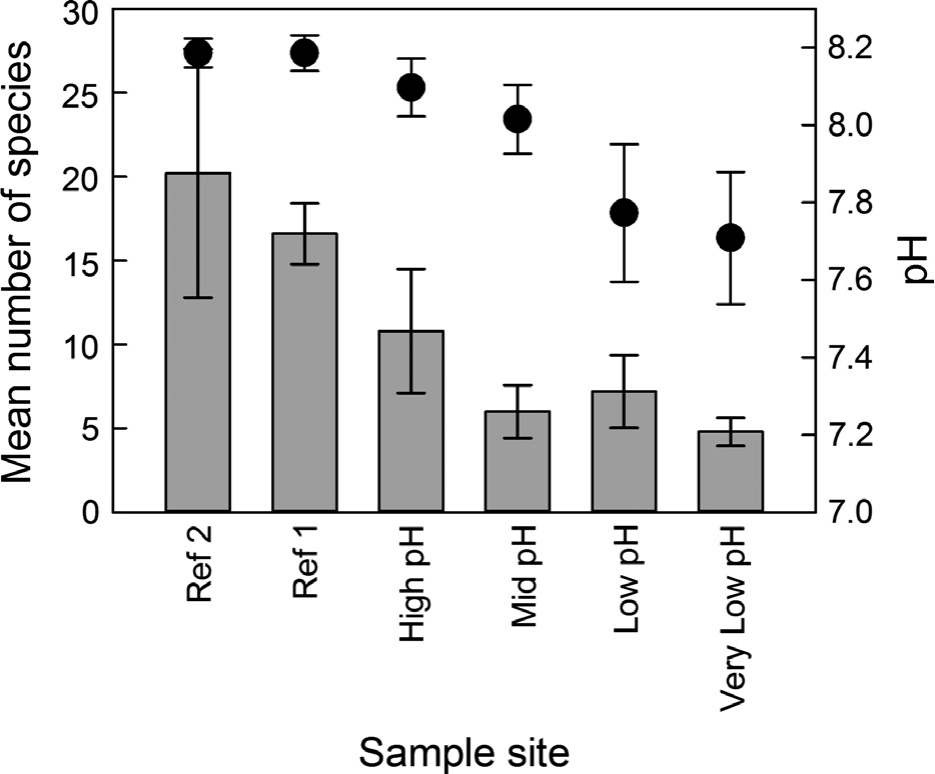
Mean (±1 SD, *n* = 5) number of species (gray bars) of epiphytic foraminifera found on *P. pavonica* thalli off Vulcano CO_2_ seeps in May 2012 with mean (±1 SD, *n* = 8–11) pH (filled circles) between May 2011 and May 2013.

Seawater calcite saturation state ranged from a mean value of ∼5.29 at reference sites to ∼2.47 extending 200 m along a rocky shore at 0–5 m water depth (Boatta et al. 2013). Mean pH_NBS_ ranged from 8.19 at the reference sites to 7.71 at the lowest pH site, which is closest to the seeps (Fig.2). The pH decreased across the gradient from reference sites to sample site very low pH (Table1) and was lower and more variable, near to the seeps.

**Table 1:** Seawater pH and associated carbonate chemistry parameters measured between May 2011 and May 2013 at six sample sites off Vulcano, Italy. Carbonate chemistry parameters were calculated from pH_NBS_ and mean total alkalinity (TA) measurements at each site (*n* values for each are shown next to site names)

Site	pH (NBS)	TA (*μ*mol/kg SW)	*p*CO_2_ (*μ*atm)	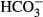 (*μ*mol/kg SW)	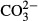 (*μ*mol/kg SW)	Ω_Calc_	Ω_Arag_
Ref 2(*n*_pH_ = 11, *n*_TA_ = 7)							
Min	8.12	2320.5	396.3	1895.7	198.7	4.66	3.10
Median	8.17	2538.7	418.0	1973.4	216.6	5.05	3.29
Max	8.25	2607.2	643.8	2023.3	250.8	5.90	3.95
Ref 1(*n*_pH_ = 10, *n*_TA_ = 7)							
Min	8.12	2368.4	395.3	1906.4	193.6	4.54	3.02
Median	8.17	2554.6	423.1	1991.8	220.9	5.15	3.36
Max	8.25	2645.6	682.4	2061.6	256.5	6.01	4.06
High pH(*n*_pH_ = 8, *n*_TA_ = 7)							
Min	7.98	2368.4	444.9	2026.5	157.7	3.69	2.44
Median	8.15	2580.3	698.6	2034.8	210.7	4.91	3.20
Max	8.15	2702.5	1292.7	2166.5	215.7	5.12	3.49
Mid-pH(*n*_pH_ = 8, *n*_TA_ = 6)							
Min	7.90	2392.4	519.0	2042.7	136.9	3.23	2.17
Median	8.01	2639.8	750.7	2193.1	167.0	3.92	2.60
Max	8.18	2776.3	1317.9	2267.7	228.8	5.36	3.54
Low pH(*n*_pH_ = 8, *n*_TA_ = 7)							
Min	7.61	2454.8	770.3	2269.9	76.0	1.77	1.15
Median	7.78	2652.6	1676.6	2461.0	114.6	2.70	1.81
Max	8.06	3004.1	2092.1	2552.0	193.7	4.54	3.00
Very Low pH(*n*_pH_ = 8, *n*_TA_ = 7)							
Min	7.61	2405.5	615.3	2182.3	77.9	1.82	1.18
Median	7.67	2663.3	1831.9	2477.0	85.9	2.01	1.34
Max	8.11	2958.3	2658.6	2495.6	207.8	4.86	3.20

**Figure 3:**
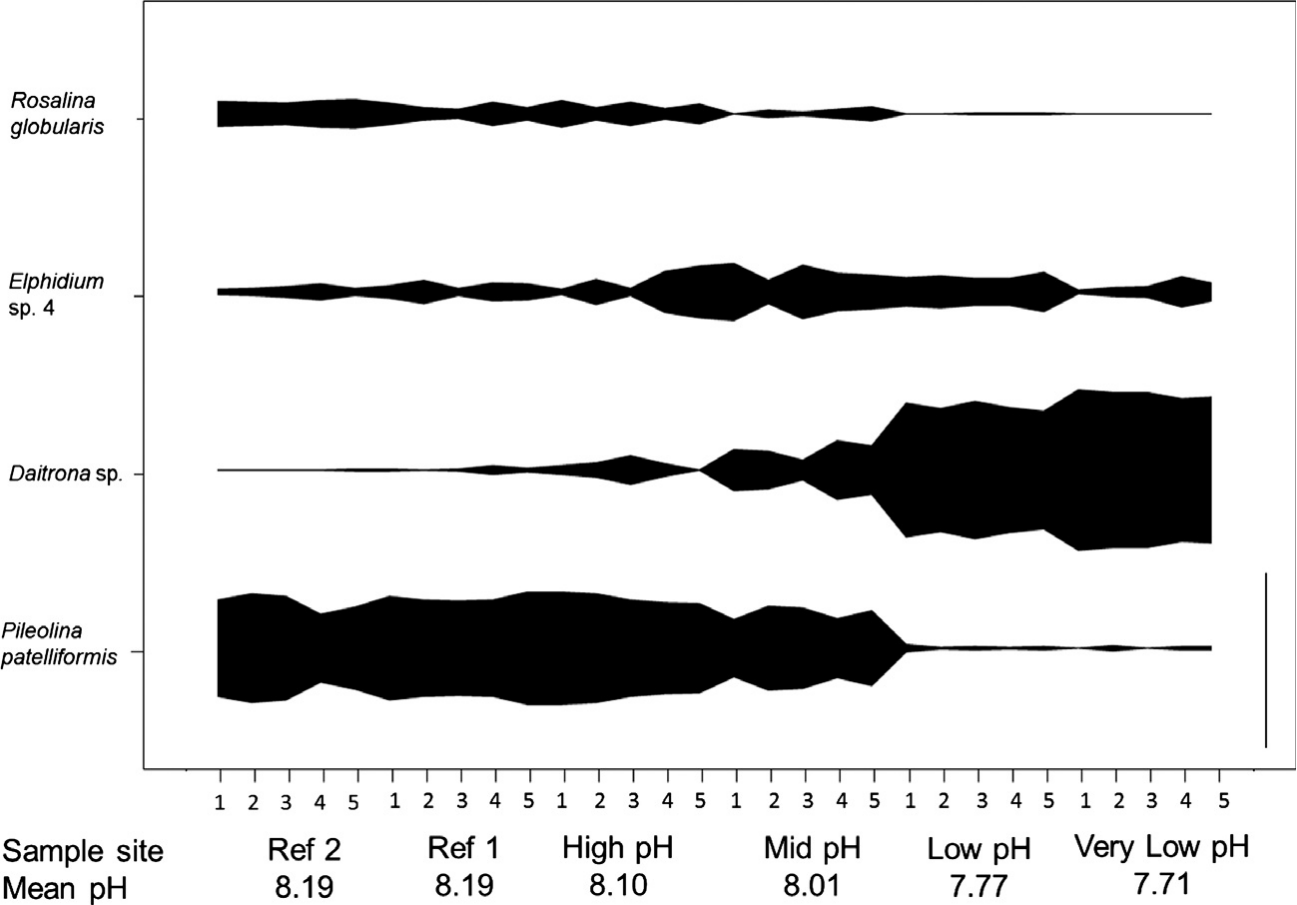
Relative abundance (%) of the four most abundant types of epiphytic foraminifera found on 2 g of dried *P. pavonica* samples at Vulcano in May 2012. Numbers 1–5 are replicate numbers. The vertical scale bar on the bottom right-hand side of the plot represents a relative abundance of 100%.

As in previous years (Johnson et al. 2012), dense stands of *Padina pavonica* characterized the rocky shore transect in May 2012. Epiphytic foraminifera were found on all 30 thalli examined with 3851 individuals counted during this study. We found a reduction in the number of species of epiphytic foraminifera along a calcium carbonate saturation gradient from reference sites (Ω ∼ 5.29) to high CO_2_ conditions (Ω ∼ 2.47) nearer to the seeps (Fig.2). The number of species ranged from 4 to 30 per replicate. Shannon–Wiener diversity ranged from 2.29 at the reference sites to 0.45 in samples collected from the most acidified site, with corresponding Fisher alpha and Pielou’s evenness indices of 10.07 to 0.88 and 0.67 to 0.28, respectively, as the most acidified samples were dominated by just a few species of foraminifera. The non-parametric Kruskal–Wallis test revealed a statistically significant difference in the number of species between sites (*P* ≤ 0.001, *H *=* *22.663, degrees of freedom = 5). The number of individuals per replicate ranged from 22 to 457 with the lowest number of individuals occurring at sites with intermediate pH. Kruskal–Wallis one-way analysis of variance on ranks revealed a statistically significant difference in the number of individuals between sites (*P * ≤ 0.001, *H* = 22.002, degrees of freedom = 5). The most abundant species was *Pileolina patelliformis* (Brady) with a total of 1541 individuals (40% of total) (Fig.3). The relative abundance of *P. patelliformis* decreased across the gradient from approximately 56.5% at one of the reference sites to 1.5% closest to the CO_2_ seeps. The next most abundant species was *Daitrona* sp. with 875 individuals (23% of total). The relative abundance of *Daitrona* sp. increased across the gradient from 0.3% at reference sites to 85.5% at high CO_2_. The assemblages were dominated by calcareous forms at reference sites (pH ∼8.19) and by agglutinated forms nearer to the seeps (pH ∼7.71). The dominant taxa in the low-pH conditions were *Daitrona* sp., which has an agglutinated test, and *Elphidium* spp.

**Figure 4:**
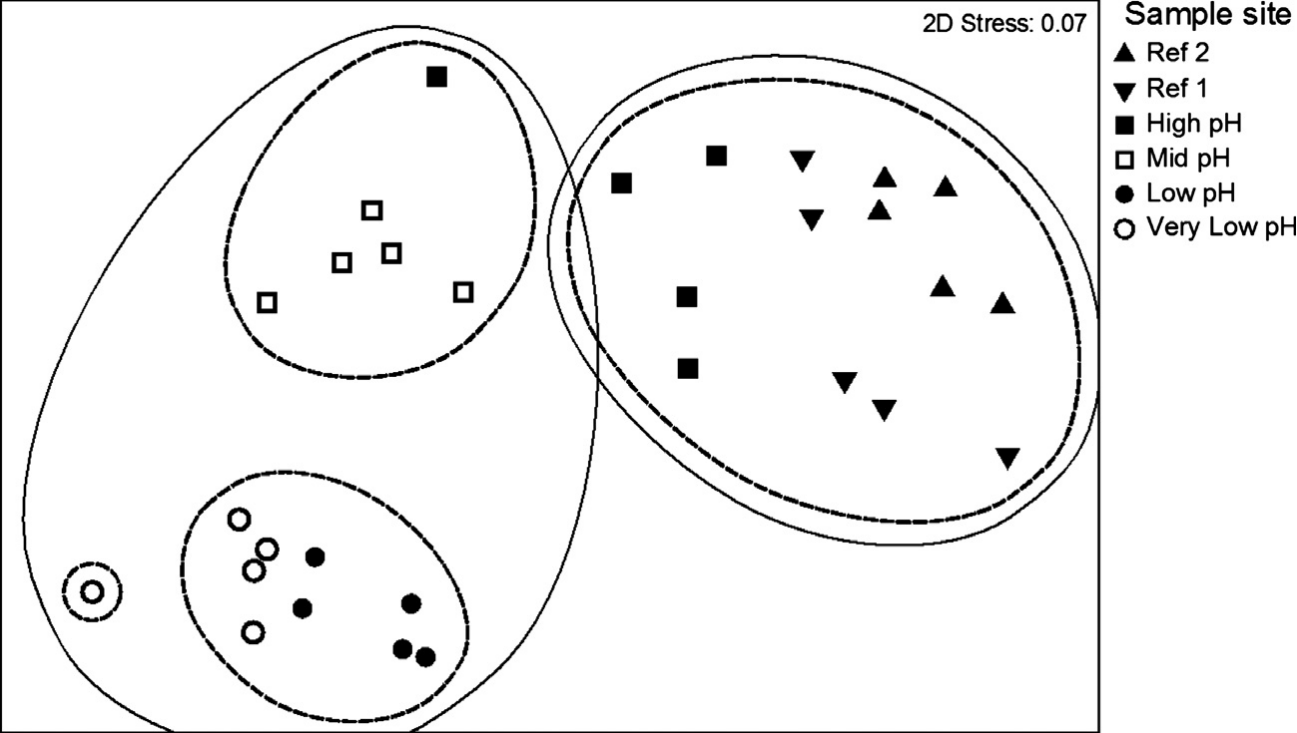
Similarities between epiphytic foraminiferal assemblages growing on *P. pavonica* thalli in 30 samples collected in May 2012 along a CO_2_ gradient off Vulcano. The circles group samples at the 40 (solid) and 60 (dashed) percent similarity levels.

Foraminiferal assemblages were similar at sample sites with similar CO_2_ levels. The sample sites cluster into four distinct groups on an nMDS plot, those from the reference sites and highest pH areas (Ref 2, Ref 1 and high pH), those from the mid-pH area with one sample from high pH, those from lowest pH areas (low pH and very low pH), and one sample from very low pH which plots as a singlet (Fig.4). One-way ANOSIM test also showed significant site differences in the assemblage (Global *R* statistic = 0.837, *P *=* *0.001). Pairwise tests show which sites were responsible for the differences. The sites that did not have a statistically significant difference in assemblage at the 0.01 significance level were as follows: Ref 2 and Ref 1; Ref 1 and high pH; and high pH and mid-pH. The assemblage of foraminifera living on *Padina* seaweed surfaces was similar between the two reference sites, a reference site and the high-pH site and the high-pH and mid-pH site. SIMPER revealed that the five taxa that contributed most to dissimilarity between sample sites were as follows: *Pileolina patelliformis*, plastogamous pairs of *P. patelliformis*, *Daitrona* sp., *Rosalina globularis* d’Orbigny, and *Peneroplis pertusus* (Forskål).

**Figure 5:**
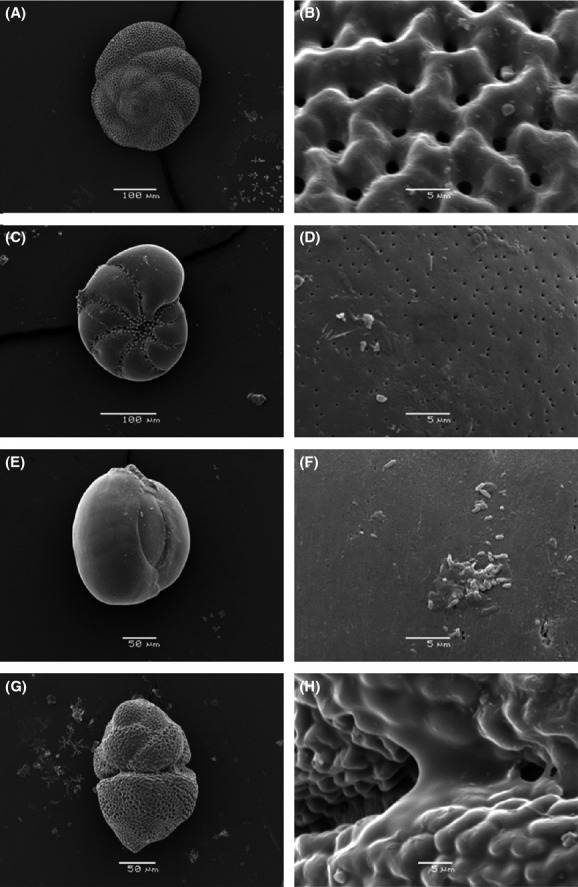
Wall structure of selected epiphytic foraminifera collected on *P. pavonica* thalli off Vulcano in May 2012. (A) *Pileolina patelliformis* from site Ref 2 (pH ∼8.19), (B) wall detail of final chamber; (C) *Haynesina depressula* from site Ref 1 (pH ∼8.19), (D) wall detail of final chamber; (E) *Miliolinella subrotunda* from site Ref 2 (pH ∼8.19), (F) wall detail of final chamber; and (G) a plastogamous pair of *P. patelliformis* from site Ref 2 (pH ∼8.19), (H) detail of the join between the two specimens.

In total, 46 individuals were examined using a scanning electron microscope (SEM) (24 from Ref 2, eight from Ref 1, three from high pH, three from mid-pH, six from low pH, and two from very low pH). Of the 11 that were examined at a higher magnification (×4000), only one showed signs of dissolution. This specimen, identified as *Miliolinella dilatata* (d’Orbigny), showed signs of surface pitting and was collected from the reference site, Ref 2. There were no deformities (such as abnormally shaped chambers) in any of the 46 individuals examined under the SEM (Fig.5), although there were a very small number of individuals (ten individuals, amounting to <1% of the total assemblage) that were noted to have deformities when examined under the light microscope.

## Discussion

As photosynthetic activity of marine plants and algae increases seawater pH (Semesi et al. 2009; Manzello et al. 2012; Hendriks et al. 2014), we predicted that seaweed growth would protect small calcareous organisms from acidified conditions near to CO_2_ seeps by raising calcium carbonate saturation state in the DBL. In fact, we found major losses of calcified foraminifera in high CO_2_ conditions. Saderne and Wahl (2013) found that fucoid algal epiphytes were resistant to *p*CO_2_ levels *ca*. 1200 *μ*atm, but at around 3150 *μ*atm, the tube worms (*Spirorbis spirorbis*) had reduced growth and settlement rates, although calcified (*Electra pilosa*) and non-calcified (*Alcyonidium hirsutum*) bryozoans were not impacted. They also argue that photosynthesis of the fucoid algae modulates calcification of small epiphytes inhabiting the algal DBL.

The degree of buffering will depend on hydrodynamics and structural characters of seagrasses or macroalgal stand (Cornwall et al. 2014; Hendriks et al. 2014). When photosynthetic biomass is high, these habitats can experience pH values above that of ambient seawater, enhancing calcification rates of associated organisms (Semesi et al. 2009). The standing stock of *P. pavonica* was clearly insufficient at Vulcano to allow a similar foraminiferal community to develop at the low-pH sites compared to the reference sites. *Padina pavonica* may buffer the pH in the DBL during the daytime, but algal respiration at night causes large daily fluctuations in pH (Cornwall et al. 2013) and it may be periodic exposure to corrosive water that excluded some of the small calcified organisms. Light levels are expected to have a strong influence on pH in the DBL (Cornwall et al. 2013). Epiphytes already experience a large daily range in pH due to light–dark cycles and fluctuations in light levels (Cornwall et al. 2013). This large daily variation may make them more resilient to future changes in pH due to ocean acidification (Cornwall et al. 2013).

The present study found a shift in the community assemblage of epiphytic foraminifera as pH decreased along a natural CO_2_ gradient from one dominated by *Pileolina patelliformis* and other calcareous taxa to one dominated by the agglutinated *Daitrona* sp. This shift occurred where mean pH reduced from ∼ 8.01 to ∼ 7.77 with a change in the mean Ω_Calc_ from 4.06 to 2.89. Distinct shifts in community assemblages have been identified before at other shallow-water CO_2_ seeps (Hall-Spencer et al. 2008; Dias et al. 2010; Fabricius et al. 2011). An ecological shift was found at a mean pH of 7.8–7.9 at Ischia (Hall-Spencer et al. 2008), and no calcareous foraminifera were found below a mean pH of ∼7.6 at Ischia, where agglutinated foraminifera dominated the foraminiferal assemblage (Dias et al. 2010). At seeps off Papua New Guinea, no foraminifera were found at sites with a mean pH below ∼7.9, and even in locations with a higher pH (∼8.0), many foraminiferal tests were corroded or pitted (Fabricius et al. 2011).

In this study, only two miliolid (porcelaneous) foraminifera were found at the lowest pH site where there was a large increase in the proportion of agglutinated foraminifera. Miliolids generally have high-magnesium calcite tests (Bentov and Erez 2006) which is more soluble than aragonite (Morse et al. 2006) making them even more vulnerable to the effects of ocean acidification. Agglutinated foraminifera are expected to be more resilient to ocean acidification, and may even benefit from the loss of calcified competitors, as they do not produce calcium carbonate tests. The materials used to construct their test, however, may still be cemented together by calcite (Sen Gupta 1999). *Daitrona* sp., which was the only agglutinated taxon present in our samples, attach sediment particles to a proteinaceous or non-calcite matrix (Sen Gupta 1999). *Elphidium* spp. were also common on *P. pavonica* in high CO_2_ conditions. The presence of *Elphidium* spp. in the low calcite saturation area suggests that they were able to calcify and maintain their calcium carbonate tests, although a small percentage (1%) was deformed. This taxon is stress tolerant (Alve 1995; Frontalini and Coccioni 2008) and has low-magnesium calcite tests (below 4 mol % MgCO_3_) (Bentov and Erez 2006), which may explain their ability to survive in high CO_2_, low calcite saturation conditions.

Although it is possible that specimens had died, but remained on the thalli after drying (Poag 1982), any epiphytic foraminifera that were attached to the thalli must have been living recently in order to attach themselves. Any that had been washed onto the thalli as dead individuals are likely to have been washed off during collection of the samples. Langer (1993) counted individuals that were still attached to the thalli as living. *Padina pavonica* is thought to have a perennial life cycle, but dies back in the winter and, therefore, any attached foraminifera cannot have been on the thalli for longer than 1 year, and the CO_2_ gradient off Vulcano is known to remain consistent across years (e.g., Johnson et al. 2012; Boatta et al. 2013) so any foraminifera on the thalli are expected to have been subjected to the same carbonate chemistry conditions as recorded in this study.

*Padina pavonica* may provide refugia from ocean acidification for certain calcified foraminifera, but the majority were unable to tolerate the most acidified conditions. Dense seagrass beds or seaweed farms may mitigate ocean acidification if the carbon produced by photosynthesis is not released back into the water column. In an examination of epibionts (coralline algae, bryozoans, and hydrozoans) on seagrass blades, Martin et al. (2008) also found a dramatic reduction in the amount of calcareous epiphytes as pH reduced, close to shallow-water CO_2_ seeps at Ischia. Below a mean pH of 7.7, bryozoans were the only calcareous epibionts present on the seagrass blades. This indicates that seagrass meadows cannot protect the full suite of calcareous epibionts from the effects of low-pH conditions.

Vizzini et al. (2013) warn against relating biological changes at volcanic CO_2_ seeps solely to pH, and it is likely that multiple drivers were affecting foraminiferal community composition across the sampling gradient. Vizzini et al. (2013) found trace element enrichment along the *p*CO_2_ gradient at Vulcano and warn that this could be a driver of any biological changes seen. Although this is a possibility, the finding of similar ecological patterns from studies at other CO_2_ seeps suggests that CO_2_ is likely to be the main environmental driver at this site. Boatta et al. (2013) argue that the northern part of the bay (the area from which samples were taken in the present study) is well suited to studies of the effects of increased CO_2_ levels.

We found a reduction in the number of species of epiphytic foraminifera on the brown seaweed *P. pavonica* along a shallow-water *p*CO_2_ gradient. There was an assemblage shift from domination by calcareous taxa at reference sites (*p*CO_2_ ∼ 470 *μ*atm) to domination by agglutinated taxa near to the seeps (*p*CO_2_ ∼ 1860 *μ*atm). The hypothesis that algal surfaces would provide a refugia for assemblages of calcified organisms along a gradient of overlying seawater acidification was not supported. It is expected that ocean acidification will result in changes in foraminiferal community composition and agglutinated forms may become more prevalent.
